# Novel Techniques in Fractional Skin Replacement

**DOI:** 10.3390/ebj6010013

**Published:** 2025-03-06

**Authors:** Courtney Kelly, Rodney K. Chan, Anders H. Carlsson

**Affiliations:** 1 Oral and Maxillofacial Surgery, San Antonio Military Medical Center, San Antonio, TX 78216, USA; 2 US Army Burn Center, San Antonio Military Medical Center, San Antonio, TX 78216, USA; chan@metisfoundationusa.org; 3 The Metis Foundation, San Antonio, TX 78216, USA

**Keywords:** cutaneous wound, partial thickness burn, full thickness burn, split thickness skin graft, full thickness skin graft, fractional autologous skin graft, expansion ratio, micrograft, RECELL, RECELL-GO, Xpansion, pixel graft, full thickness skin column, ART device

## Abstract

The gold standards for coverage of wounds that cannot be primarily closed are full thickness skin grafts (FTSGs) and split thickness skins graft (STSGs). FTSGs harvest sites generally require primary closure, which limits availability, especially when treating larger wounds. STSGs have many shortcomings, including donor site morbidity. Fractional autologous skin replacement can be utilized in conjunction with or in lieu of STSGs to both improve graft outcomes of large wounds and to decrease donor site morbidity. Skin can be mechanically or chemically fractionated. Fractionated skin can be advantageous, as adnexal structures provide additional functionality without donor site morbidity. In this review, we will discuss current and emerging techniques in fractional skin replacement.

## 1. Introduction

Critical-size skin defects often result either in the inability of the body to appropriately close an open wound or scarring that impairs function, both of which require surgical intervention. Surgical treatment modalities for skin defects commonly include full thickness skin grafts (FTSGs) and split thickness skin grafts (STSGs). Typically, STSGs are utilized for larger and sub-optimal wounds whereas FTSGs are indicated for smaller and well-optimized wounds, as thicker grafts contain more biological elements and thus more requirements for ensuring their survival [1].

Fractional skin replacement is based on the concept that an autologous, non-cultured graft can be fractionated by either mechanical or chemical means to cover large defects while decreasing the harvest site size and, therefore, morbidity. Patient and wound selection is critical when determining which grafting technique to employ. FTSGs and STSGs require strict immobility and maintenance of the fragile graft elements to minimize shear and traction, ensuring survival.

In this review, we will focus on current and emerging techniques in the field of fractional skin replacement. We will not discuss cultured or traditional tangentially-harvested dermal/epidermal autografts, nor off-the-shelf and/or tissue-engineered skin substitutes. The following are the device systems which will be further presented in this review.

RECELL^®^, originally developed in the late 1980s and approved for use by the FDA in 2018, works by chemically processing an autologous STSG to produce a cell suspension that is sprayed onto an open wound [2,3,4]. The Xpansion Micrografting System, launched in 2010, mechanically cuts micro-grafts, enabling an expansion ratio of up to 1:100 [5,6,7,8]. Full thickness skin columns (FTSCs), a method developed in 2012, mechanically obtains full thickness micro-grafts, bringing the functional adnexal structures to the wound bed [9,10,11,12,13]. We will elaborate on these methods and supporting research. We will also propose ideal candidates (patient and wounds) to employ these modalities, based on their unique characteristics, in comparison to the standard of care for FTSGs and STSGs.

## 2. Principles of Fractional Skin Replacement

Skin grafting itself is not a novel concept; there is evidence of skin grafting practices in Ancient Egypt, c. 3000 BC, and by Hindu surgeons, c. 800 BC [14,15]. FTSGs and STSGs both became well documented in the mid-to-late-1800s [14,15]. To further discuss the advances of skin grafting, it is critical to understand our current gold standards. What are the benefits and pitfalls of the full and split thickness grafts?

Wolfe in 1875 and Krause in 1893 are together credited with the advent of the dermo-epidermal (full thickness skin) graft [15]. FTSGs typically have more resemblance to normal skin texture, elasticity and suppleness, due to its abundance of dermis and the presence of adnexal structures, and are therefore commonly utilized in cosmetically important areas such as the maxillofacial region [16,17]. Donor sites for FTSGs are typically primarily closed, and therefore have low morbidity. The post-operative period for a FTSG must involve compression, often using a bolster that is removed at one week; additionally, the area should be immobilized, which may require the fabrication of a splint, depending on the site [1]. Inosculation is complete for FTSGs by 1 week and reinnervation can take years before returning to normal [17].

**Figure 1:**
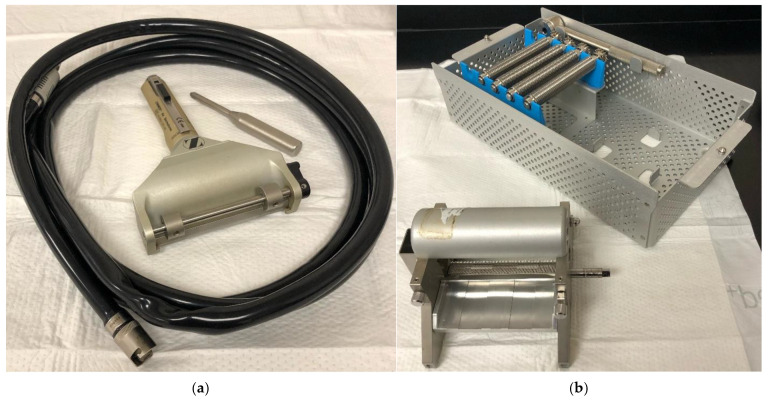
(**a**) Zimmer Air Dermatome. (**b**) Zimmer Skin Graft Mesher System, which offers different expansion ratios. Photos captured by the authors.

Conceptualized by Janzekovic in 1970, the present-day STSGs are typically reserved for larger wounds [14]. Various thicknesses can be obtained by pneumatic dermatome and then meshed at varying expansion ratios, often ranging from 1:1 to 3:1 (Figure 1) [5,18]. Increasing the expansion ratio higher than 1:1 will allow for a smaller donor graft in comparison to the recipient site. Zimmer Biomet offers a mesh expansion system that achieves expansion at a 9:1 ratio. When patients have less healthy available skin, such as in the case of Total Body Surface Area (TBSA) > 30%, expansion ratios will commonly be at least 2:1 [19]. The healing time is affected by the expansion ratio. A 1:1 ratio will heal fastest, and a 6:1 ratio will heal in 17 days, as there is immediate epithelial coverage resulting in faster inosculation. Increasing the expansion ratio results in longer healing time due to increased epithelial migration distances, decreased cellular density, and greater requirement for neovascularization [20]. The post-operative period for a STSG typically involves dressing the surgical site, possibly with negative pressure wound therapy (NPWT) or with a bolster dressing that remains in place for usually four to seven days to prevent mobility, shear forces, desiccation, or fluid accumulation [21,22]. STSGs are typically adherent after 1 week and graft maturation can last for years [16].

There are unappealing qualities belonging to the STSG. For instance, the donor or harvest site for STSGs can result in scar hypertrophy, pain, pruritis, hyperpigmentation, and possible infection [15,23]. When these harvest sites result in unesthetic or uncomfortable hypertrophic scarring, patients may be treated as needed with a secondary procedure, such as laser therapy or surgical intervention. Donor site morbidity is decreased to a degree by meshing, as a smaller donor site is required for a larger wound bed. However, the meshing often results in an unsightly “fishnet” appearance [5]. The graft itself lacks deep dermal adnexal structures, such as hair follicles and sweat glands, and is therefore lacking in complete functionality [24]. The STSG may require a preceding acellular dermal matrix if the wound bed is not yet adequately vascularized, which would take up to 2–3 weeks prior to completion of the STSG, adding significant overall healing time for patients [16].

Fractional Skin Replacement utilizes the principle of epithelial migration from graft edges to accelerate wound closure. Therefore, multiple smaller grafts provide numerous active edges for regeneration, increased epithelial surface area, and increased epithelial migration, which then results in quicker re-epithelization and a more esthetic graft site. However, higher expansion ratios may prolong healing times and affect functional outcomes, due to the reduced density of transplanted dermal structures [5,24,25,26].

Micrografting was originally documented in 1958 with the Meek Wall dermatome, which cut grafts into 4 mm × 4 mm postage stamp-shaped split thickness micro-grafts with expansion ratios of 4:1 to 9:1 [5,14,27]. This technique passed the harvested graft twice on a cork plate carrier through the 13-blade dermatome, then a coat of skin glue was sprayed onto the micro-grafts in order to transfer them to the wound bed [26,27]. Success with this technique has been demonstrated at 75–90% [5,14]. The main pitfall of this technique is difficulty in handling the micro-grafts and the possible requirement of dermal orientation for graft survival, which will be discussed further below [5,26]. A modernized Meek micrograft was re-engineered in 1993 by Kreis et al. through Humeca Medical. This modern Meek machine similarly uses a cork plate carrier to pass a 13-blade dermatome twice through the STSG, creating 196 split thickness micro-grafts measuring 3 mm × 3 mm each, which are then transferred via adhesive spray to a sterile adherent gauze (Plissee). The Plissee is stretched and unfolded, creating the intended expansion, and is able to be easily transplanted to the recipient site while maintaining appropriate dermal orientation [28].

Dermal orientation refers to the process of a graft being placed with the dermal side in contact with the wound bed. It is a labor- and time-intensive process to individually orient each micrograft properly, which deters surgeons [5,26,29]. Recent studies in fractional skin replacement have demonstrated that micro-grafts can survive without appropriate dermal orientation when placed in a moist environment [5,30,31]. Typically, polyurethane wound chambers are used to provide moist incubator-like environments for the micro-grafts. Histological studies demonstrate that keratinocytes proliferate from the border edges and basal layer of split thickness micro-grafts, creating a new basement membrane within 6 days of transplantation and a continuous dermal epidermal junction soon after [30].

To provide a clearer understanding of the various autologous skin grafting techniques discussed, Table 1 summarizes their key characteristics, including wound depth, harvest size, expansion ratios, donor site morbidity, reepithelization timelines, and specific clinical indications. This comparative overview highlights the strengths and limitations of each approach, from traditional methods, like FTSG and STSG, to innovative fractional techniques, such as RECELL, Xpansion Micrografting, and Full Thickness Skin Columns. These techniques are further detailed in the following sub-sections.

### 2.1. Xpansion Micrografting and Pixel Grafting

**Figure 2:**
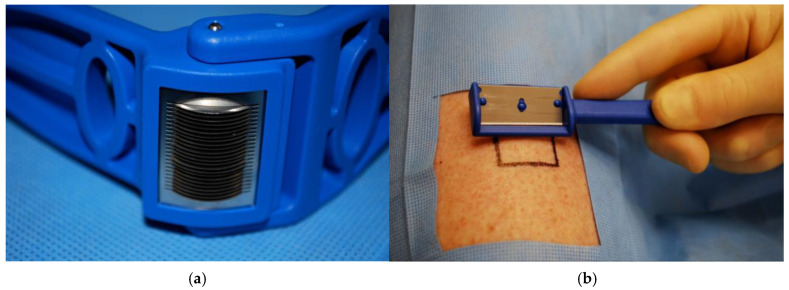
(**a**) Xpansion micro-autografting system, 24 parallel rotating cutting discs. (**b**) Clinical use of Xpansion device. Applied Tissues Technologies have the copyright to Figure 2a,b and have given the authors permission to use it in this publication.

Micrografting techniques have been modified throughout the years to increase the reliability of graft take and to increase the expansion ratio. The Xpansion^®^ Micro-autografting system (Applied Tissue Technologies, Newton, MA, USA) (Figure 2), developed by Dr. Elof Eriksson and launched in 2010, cuts grafts into 0.8 mm × 0.8 mm micro-grafts, thereby enabling an expansion ratio of 1:100 [5,6]. In preclinical trials, the expansion ratio has been demonstrated up to 500 times [7]. It is designed similarly to the Meek Wall dermatome, but with 24 parallel rotating cutting discs to create smaller micro-grafts, maximizing autologous donor tissue. The STSG is harvested and placed twice through the Xpansion mincer perpendicularly, then the micro-grafts are applied to a wound bed by scatter technique with a spatula. Post-operative care requires a moist environment over the grafted site, which eliminates the need for dermal orientation, and immobilization of the area for 10–14 days. This application is appropriate for a variety of complex wounds, including burn, traumatic, chronic, or surgical wounds, and can be completed under local anesthesia [8].

Pixel grafts are even smaller, appearing clinically as a paste and averaging 0.3 × 0.3 mm in size under a microscope. These grafts may be created using the same Xpansion mincing device, this time cuttings grafts 5 times in each perpendicular direction [5]. A dermal spray device is then used to apply the minced graft onto a wet collagen sheet, which is placed over the wound bed. Post-operative care includes a non-adhesive dressing for seven days, and average reepithelization required 30 days with an average 80% graft take. In a paper by Chavan et al. from 2021, the authors describe an advantage of pixel grafting over micrografting, in that graft survival is improved due to a decreased diffusion distance for nutrients [32,33].

These minced micrografting techniques allow for a smaller harvest site by taking advantage of epidermal migration from graft edges. While complete reepithelization takes longer than an FTSG or STSG as there is less actual donor graft tissue, there is improvement in wound contracture and mechanical stability compared to a nongrafted wound [33]. A longer post-operative period may include increased follow-up appointments for patients and possible increased time with immobilization of an extremity, depending on the wound location. Another disadvantage of Xpansion micrografting and Pixel grafting is that there is possibly poor cosmesis at the grafted sites [32]. For these reasons, areas for employment of these grafting techniques should be reserved for non-esthetic zones and non-mobile areas.

### 2.2. RECELL^®^

**Figure 3:**
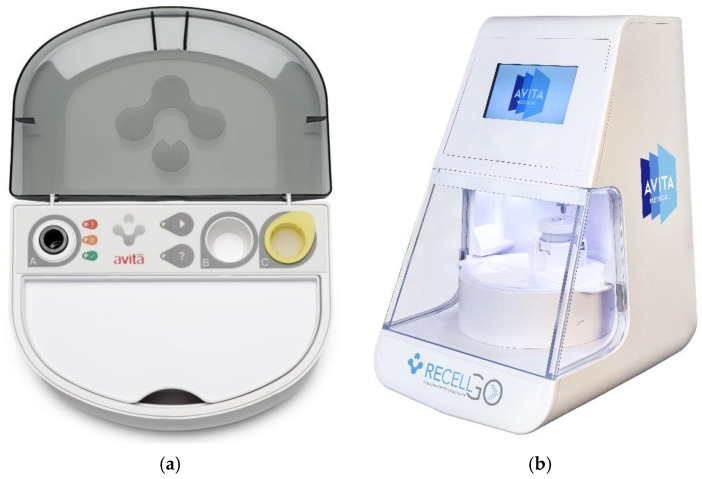
(**a**) RECELL^®^, single-use cartridge. (**b**) RECELL-GO^®^, multi-use processing device with single-use cartridge. *Avita Medical have the copyright to* Figure 3a,b *and have given the authors permission to use it in this publication*.

The RECELL^®^ Autologous Cell Harvesting Device was initially developed in the late 1980s by Dr. Fiona Wood, and originally called CellSpray, and later, Spray-on-Skin™ [15]. The RECELL System (Avita Medical, Inc., Valencia, CA, USA) (Figure 3a) was only recently approved for use in the US by the FDA in 2018 [15,34]. RECELL works by enzymatically processing a small (4–5 cm^2^) autologous STSG to produce an epidermal regenerative cell suspension that is sprayed on the open wound. This process overcomes the limitation of healing only from the wound and graft edges [35,36]. The autologous skin cell suspension includes disaggregated keratinocytes, fibroblasts, and melanocytes, which aid in re-epithelization and pigment restoration of tissue [2]. The RECELL system has also been used in combination with a widely meshed STSG to decrease donor site size and increase graft re-epithelization [3]. Indications for use include repigmentation of vitiligo patients, thermal burns, chronic wounds, and donor sites. In June 2023, RECELL earned FDA approval for treatment of full thickness skin defects, to include tissue degloving injuries and necrotizing soft tissue infection wounds.

The RECELL^®^ Autologous Cell Harvesting Device can process 24 cm^2^ donor skin into 6 mL of cell suspension, which can treat a total of 480 cm^2^ (including the 24 cm^2^ donor site). An STSG is obtained and placed with a pre-heated trypsin enzyme in the device for 15 to 20 min. The STSG is then moved to a buffered well, where the epidermis is scraped to disaggregate the cells into suspension. The Spray-On Skin™ Cell suspension is strained and then delivered to the treatment site via spraying or dripping [35]. This cell suspension delivers keratinocytes, melanocytes, and dermal fibroblasts to the wound bed. Post operative care requires a non-adherent small-pore dressing, such as a Telfa™ Clear wound dressing, for 5–8 days, then protecting for 2 weeks with Xeroform™ and compression dressings [3]. The manufacturer’s economic model reports total treatment costs using RECELL as, overall, less expensive when compared to conventional dressings, taking into account faster healing and shorter hospital stays [35]. Using RECELL and a widely expanded STSG together is clinically and economically advantageous compared to a conventional STSG [2].

The RECELL GO^®^ System (Avita Medical) is the next generation Spray-On Skin Cell device, which was recently FDA-approved in May 2024 (Figure 3b). This multi-use processing device simplifies the product, improves operating room efficiency, and has better control of the incubation time for the enzyme to optimize the cell suspension. The practitioner simply places the RECELL enzyme, buffer, and STSG into a cartridge and starts the machine, which will run for around 35 min. The processing device is reusable, while the cartridge is single use. Both RECELL and RECELL GO can process up to a 24 cm^2^ STSG at a time which may treat up to 1920 cm^2^ in area, resulting in an expansion ratio of 1:80 [2,3,35,36].

### 2.3. Full Thickness Skin Columns

Fractional autologous skin harvesting of multiple small full thickness skin columns (FTSCs) has proved to reduce donor size while improving graft site re-epithelization outcomes. While we will refer to these grafts as FTSCs in this review, they have also been addressed as microscopic skin tissue columns, micro skin tissue columns, and full thickness skin tissue columns in various articles.

In the late 1800s, Jacques-Louis Reverdin pioneered the “pinch graft,” a rudimentary version of the FTSC, i.e., small full thickness skin grafts harvested by lancets. This technique has been modified through the years and popularity has waned; however, the surgical technique remains basic, utilizing a scalpel to harvest numerous small FTSGs that are individually placed onto a wound bed [4]. While this results in expansion of an FTSG, it remains necessary primarily to close the donor site. FTSCs are a modernization of historic pinch grafts, negating the need for primary closure of the donor site. The processes of fractional photo-thermolysis helped to conceptualize modern use of the FTSC. This is a process in which laser microbeams produce “microthermal zones”, which are thin columns of thermally damaged and removed tissue [9,10]. These coring sites undergo regeneration via an increase in fibroblast activity, seen histologically with undulating rete ridges [9,10,11]. This has been well studied from the early 2000s, with fractional lasers being widely popularized for use in esthetic treatments of the aging face, as well as scar contractures. Along with this scientific theory, the assumption is that harvesting FTSCs will result in negligible morbidity of the donor site. The following articles will detail their findings on donor sites, but more research is required to confirm the negligible morbidity.

Fractional harvesting of FTSCs is demonstrated as superior to STSGs, since donor site morbidity is close to negligible, the graft site heals in a uniform pattern without “fishnet” texturing, and adnexal structures remain intact [5,12,37,38]. The FTSG has superior properties to the STSG, as dermal architecture is transferred, thereby increasing tensile strength and improving cosmesis [38]. However, the donor site of the FTSC should be primarily closed, which decreases the possibilities in size and in the locations they can be harvested from [13]. FTSCs combine properties of both FSTGs and STSGs to improve upon the structural aspects of STSGs. However, as of the latest research, FTSG still remains the gold standard in areas of cosmetic and functional concern, such as the head and neck.

In the first reported use of FTSCs in 2013, Tam et al. first harvested 700 μm FTSC on adult swine with 19 G hypodermic needles and a constructed fluidic device. The columns were placed in random orientation and healed appropriately by week 2, with the donor site clinically and histologically undetectable after week 7 [10].

More research is needed in terms of ideal expansion ratios. As expected, transplantation of a higher number of FTSCs to a wound bed will result in faster and improved re-epithelization [24,38]. The maximum harvest density determined ex vivo on swine is sixteen 1.5 mm skin columns per cm2 (28% density), accounting for intact skin bridges and no deep overlaps. At this density of harvest, the donor site heals similarly to that of an STSG [38]. At a density of 7–10% (four 1.5 mm columns per cm^2^), the donor site will be clinically and histologically indistinguishable from uninjured tissue at 60 days [38].

In a human case report completed in a 2020 publication, post-operative times are seen to be longer than for standard FTSGs or STSGs. The cases utilized 1.5 mm and 2 mm biopsy punches to harvest skin columns and implant them into the Integra bilayer dermal matrix. By day 60, the wound site was fully re-epithelialized with minimal pain or scarring. The donor sites had completely healed without scarring by day 30 [37].

### 2.4. Full Thickness Skin Columns in Development

Innovations in FTSCs are currently in development, and we should see new devices and applications within the next few years. MagneTEskin is a construct that uses iron oxide (Fe_3_O_4_) particles to retain appropriate dermal orientation during implantation of FTSCs [29]. More research is warranted to indicate the necessity for appropriate orientation when placing the FTSC into a wound bed, as the cost/benefit analysis may not prove beneficial at this time [29].

**Figure 4:**
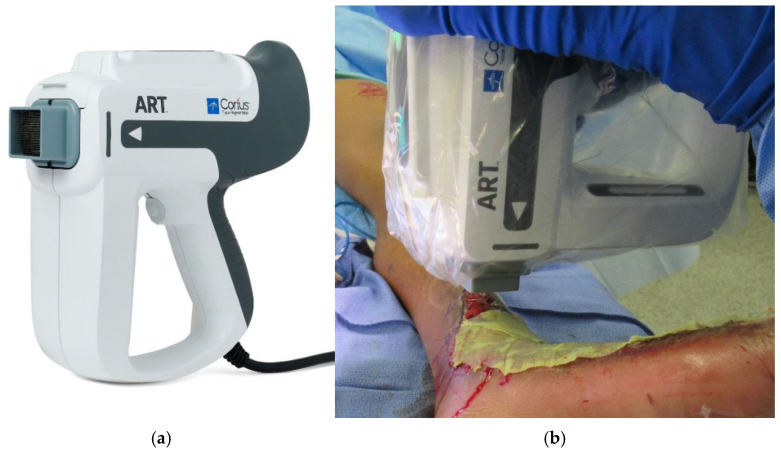
(**a**) ART Device, *Medline have the copyright to this figure and have given the authors permission to use it in this publication.* (**b**) Clinical application utilizing handheld ART device to harvest and then apply the FTSC at the wound site; *Photo captured by the author*.

The ART system (Medline Industries, Northfield, IL, USA) (Figure 4) is being developed to decrease the tedious time and labor required for harvesting and implanting the columns. ART (Autologous Regeneration of Tissue) is an automated, reusable, handheld device that is capable of harvesting FTSCs and transferring them to a wound bed with correct dermal orientation. A single cartridge can harvest up to 316 FTSCs at 500 μm wide and 3.25 mm depth, equaling approximately 10% of skin density, which, as discussed above, will typically result in negligible donor site morbidity at 60 days [38,39,40].

Another automative harvesting system currently in development is the Sperry Micro-Coring System (Sperry Bio Inc., Springfield, VT, USA), where harvested FTSCs are distributed in a fixed and regular pattern, obviating the issue of skin-stacking and contact inhibition and possibly resulting in a more regular grafted scar. Further, the Sperry Micro-Coring system also transfers skin adnexal structures.

## 3. Discussion

Fractional skin replacement modalities have proven to be useful techniques in certain wounds and patients. Wounds in non-esthetic zones that require an STSG may benefit from fractional skin replacement. STSG harvest sites can not only heal unesthetically but also may lead to discomfort and pruritus. Because of this, patients frequently require secondary procedures at their harvest sites, such as Pulse-dye, IPL, or CO2 laser therapy, and occasionally surgical intervention. Decreasing the size of the donor site will in turn decrease morbidity for the patient.

RECELL enzymatically processes an STSG into a suspension that can be applied directly to a wound either in conjunction with a heavily expanded STSG, or by itself. Xpansion micro-grafts and minced Pixel grafts mechanically processes the STSG into much smaller fragments, increasing the expansion ratio and decreasing the donor site morbidity. Re-epithelialization and healing time in micro-grafts are longer compared to FTSGs and STSGs because the reduced amount of transferred tissue necessitates greater epithelial migration. However, the increased number of epidermal edges per unit area facilitates multiple points of regeneration, partially offsetting the delayed closure. The FTSC combines properties from the FTSG and STSG to improve upon the appearance of the grafted site while decreasing the donor site morbidity. Although it can be theorized that FTSCs can be used in functional and esthetic zones, research remains to be published on the matter. FTSC harvesting devices are currently under development and will hopefully prove beneficial in easing the labor and time required.

As these harvesting devices have been engineered to help decrease labor and the difficulty of grafting, is also the possibility of the use of these devices possibly by non-surgeons. RECELL, Xpansion, and the ART harvesting devices can be utilized under local anesthesia, mitigating the requirement for operating room support. These devices may also prove to be beneficial in downrange military theatres if medical evacuation becomes unavailable. Nevertheless, its usage in this setting maybe limited by the adequacy of debridement in an austere setting.

Overall, fractional autologous skin replacement has shown promise in the treatment of open wounds without incurring much donor site morbidity. There are recent exciting advances in the field, and better understanding of how the survival of these fragile skin elements is paramount to the success of these technologies. Long-term follow-up studies are required to better assess the functional outcomes of fractional autologous grafting. A cost benefit analysis incorporating the need for additional procedures and return to function will be important to determine the feasibility of these novel products.

## Figures and Tables

**Table 1:** Comparison of Autologous Skin Grafting Techniques.

Technique	Wound Depth	Harvest Size	Expansion	Donor site Morbidity	Reepithelization/Graft Take	Common Uses
**Traditional skin grafting**						
**FTSG**	Full	Limited by closure	1:1	Primary closure	7–14 days	Smaller wounds In esthetic zone At articulating joints
**STSG**	Full	Limited by available skin	1:1–1:9	Significant	7–14 days	Large wound
**Fractional skin grafting**						
**Meek** **Micrograft**	Partial	18 cm^2^ as 5 × 5 mm each *	1:2–1:4	Moderate	14–21 days	Large wound
**Xpansion Micrograft**	Partial	4 cm^2^ as 0.8 × 0.8 mm each *	Up to 1:100	Moderate	14–21 days	Large wound
**Pixel** **Micrograft**	Partial	4 cm^2^ as 0.3 × 0.3 mm each *	Up to 1:400	Minimal	14–30 days	Large wound
**RECELL**	Partial	24 cm^2^ *	1:80	Minimal	30 days	Vitiligo, wounds, donor sites
**FTSC**	Full	15.5 cm^2^ *	1:10–1:50	Negligible	14 days 60 days (normal skin)	Vitiligo, wounds

* Indicates *total* harvest size remaining “limited by available skin”.

## Data Availability

No new data were created or analyzed in this study. Data sharing is not applicable to this article.
